# Supplementary material for: Pathophysiological Significance of Neutrophilic Transfer RNA-Derived Small RNAs in Asymptomatic Moyamoya Disease
Source: Cells. 2021 May 1;10(5):1086. doi: 10.3390/cells10051086 (PMC8147334; doi:10.3390/cells10051086)
Supplement: Supplementary file 1 [file cells-10-01086-s001.zip › cells-1149161 3rd supplementary materials/Data Supplements/Data Supplement V.zip!/Data supplement 6-mRNAs targets of tsRNAs/tsRNA-Glu-TTC-010/Diagrams/tRF-Glu-TTC-010_vs_ENST00000399604(ZNF594).pdf]

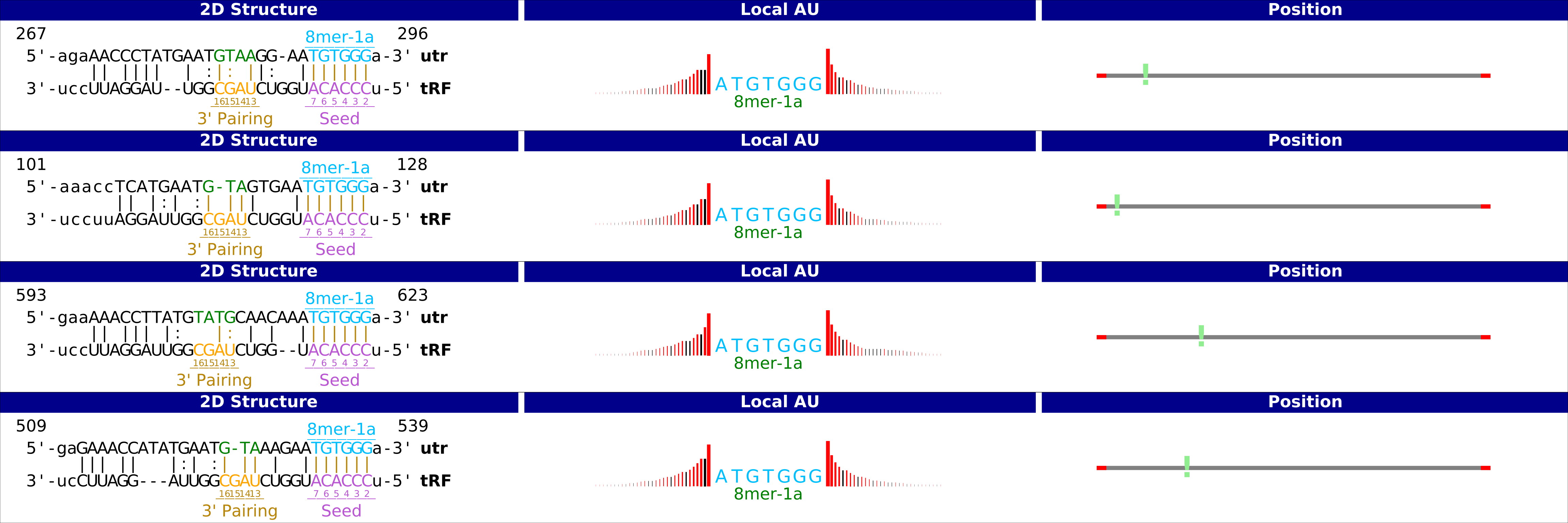

2D Structure
Local AU
Position
267
8mer-1a
296
5'-agaAACCCTATGAATGTAAGG-AATGTGGGa-3' utr
3'-uccUUAGGAU--UGGCGAUCUGGUACACCCu-5' tRF
16151413
7 6 5 4 3 2
3' Pairing
Seed
ATGTGGG
8mer-1a
2D Structure
Local AU
Position
101
8mer-1a
128
5'-aaaccTCATGAATG-TAGTGAATGTGGGa-3' utr
3'-uccuuAGGAUUGGCGAUCUGGUACACCCu-5' tRF
16151413
7 6 5 4 3 2
3' Pairing
Seed
ATGTGGG
8mer-1a
2D Structure
Local AU
Position
593
8mer-1a
623
5'-gaaAAACCTTATGTATGCAACAAATGTGGGa-3' utr
3'-uccUUAGGAUUGGCGAUCUGG--UACACCCu-5' tRF
16151413
7 6 5 4 3 2
3' Pairing
Seed
ATGTGGG
8mer-1a
2D Structure
Local AU
Position
509
8mer-1a
539
5'-gaGAAACCATATGAATG-TAAAGAATGTGGGa-3' utr
3'-ucCUUAGG---AUUGGCGAUCUGGUACACCCu-5' tRF
16151413
7 6 5 4 3 2
3' Pairing
Seed
ATGTGGG
8mer-1a
